# Hydroxyapatite-decorated Fmoc-hydrogel as a bone-mimicking substrate for osteoclast differentiation and culture

**DOI:** 10.1016/j.actbio.2021.11.011

**Published:** 2022-01-15

**Authors:** Mattia Vitale, Cosimo Ligorio, Bethan McAvan, Nigel W. Hodson, Chris Allan, Stephen M. Richardson, Judith A. Hoyland, Jordi Bella

**Affiliations:** a Division of Cell Matrix Biology and Regenerative Medicine, School of Biological Sciences, Faculty of Biology, Medicine and Health, The University of Manchester, Manchester M13 9PT, United Kingdom; b BioAFM Facility, Faculty of Biology, Medicine and Health, The University of Manchester, Manchester, United Kingdom; c Biogelx Ltd-BioCity Scotland, Bo'Ness Rd, Newhouse, Chapelhall, Motherwell ML1 5UH, United Kingdom

**Keywords:** Osteoclast culture, Bone tissue engineering, Peptide hydrogel, Hydroxyapatite, Macrophage

## Abstract

Hydrogels are water-swollen networks with great potential for tissue engineering applications. However, their use in bone regeneration is often hampered due to a lack of materials’ mineralization and poor mechanical properties. Moreover, most studies are focused on osteoblasts (OBs) for bone formation, while osteoclasts (OCs), cells involved in bone resorption, are often overlooked. Yet, the role of OCs is pivotal for bone homeostasis and aberrant OC activity has been reported in several pathological diseases, such as osteoporosis and bone cancer. For these reasons, the aim of this work is to develop customised, reinforced hydrogels to be used as material platform to study cell function, cell-material interactions and ultimately to provide a substrate for OC differentiation and culture. Here, Fmoc-based RGD-functionalised peptide hydrogels have been modified with hydroxyapatite nanopowder (Hap) as nanofiller, to create nanocomposite hydrogels. Atomic force microscopy showed that Hap nanoparticles decorate the peptide nanofibres with a repeating pattern, resulting in stiffer hydrogels with improved mechanical properties compared to Hap- and RGD-free controls. Furthermore, these nanocomposites supported adhesion of Raw 264.7 macrophages and their differentiation in 2D to mature OCs, as defined by the adoption of a typical OC morphology (presence of an actin ring, multinucleation, and ruffled plasma membrane). Finally, after 7 days of culture OCs showed an increased expression of TRAP, a typical OC differentiation marker. Collectively, the results suggest that the Hap/Fmoc-RGD hydrogel has a potential for bone tissue engineering, as a 2D model to study impairment or upregulation of OC differentiation.

**Statement of significance:**

Altered osteoclasts (OC) function is one of the major cause of bone fracture in the most commonly skeletal disorders (e.g. osteoporosis). Peptide hydrogels can be used as a platform to mimic the bone microenvironment and provide a tool to assess OC differentiation and function. Moreover, hydrogels can incorporate different nanofillers to yield hybrid biomaterials with enhanced mechanical properties and improved cytocompatibility. Herein, Fmoc-based RGD-functionalised peptide hydrogels were decorated with hydroxyapatite (Hap) nanoparticles to generate a hydrogel with improved rheological properties. Furthermore, they are able to support osteoclastogenesis of Raw264.7 cells *in vitro* as confirmed by morphology changes and expression of OC-markers. Therefore, this Hap-decorated hydrogel can be used as a template to successfully differentiate OC and potentially study OC dysfunction.

## Graphical abstract

**Figure:**
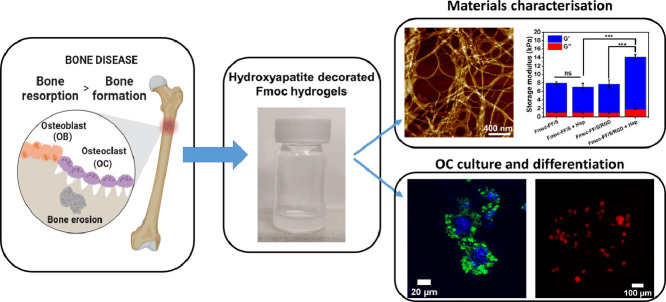

## 1 Introduction

Bone is a highly dynamic and hierarchically organised organ, consisting of a complex network of cells embedded in a 3D structure of organic-inorganic nanocomposite, which confers remarkable mechanical performance, including high strength and fracture toughness [1,2]. Bone is constantly self-renewing through a finely regulated balance of two processes: osteoclastogenesis, through bone-resorbing osteoclasts (OCs), and osteoblastogenesis through bone-forming osteoblasts (OBs) [3], [4], [5]. Disruption of the equilibrium between these processes can cause bone defects or may lead to increased risk of bone fractures [6]. OCs are hematopoietically-derived multinucleated cells that play an essential role in maintaining bone turnover and they are key players in bone diseases, such as osteoporosis and different forms of cancer [5,7,8]. Bone tissue engineering can provide useful tools to mimic the natural bone microenvironment and study the mechanisms involved in bone diseases. Synthetic or natural biomaterial scaffolds have been used to promote the migration, proliferation, and differentiation of pluripotent cells into bone cells [9,10]. Hydrogels are water-swollen polymer-based networks, synthesized from hydrophilic monomers, which are able to form self-supporting systems that do not dissolve in water [11]. Hydrogel-based scaffolds are currently used for bone studies because they can provide a realistic hydrophilic 3D environment that supports cell attachment, spreading and new bone ingrowth [12], [13], [14]. However, a successful scaffold for bone regeneration should take into account the complexity of the bone turnover and provide biomaterials that can regulate OC activity accordingly [15]. Yet, very little is known about the interaction of OCs and biomaterials.

Self-assembling peptide-based hydrogels have shown physicochemical features mimicking native extracellular matrix (ECM), due to their high percentage of water (> 90% of the dry weight), tuneable mechanical properties and nanofibrous architecture [16], [17], [18], [19]. They also offer multiple avenues for the design of bioactive materials by incorporating natural motifs for the dynamic control of the materials final structures and their interactions with cells [20]. It has been demonstrated that aromatic short peptides are able to form stable peptide nanotubes through a combination of hydrogen-bonding and π-stacking interactions [21]. In particular, the chemical coupling of aromatic protecting groups such as 9-fluorenylmethoxycarbonyl (Fmoc) to the N-terminus of some peptides helps them to spontaneously assemble into self-supporting hydrogels [22]. Amongst Fmoc-functionalised peptides, Fmoc-diphenylalanine (Fmoc-FF) has been demonstrated as an effective low molecular weight gelator, forming a rigid cylindrical-shape dipeptide hydrogel without the need for additional crosslinking agents [23]. Additionally, the chemical properties of these hydrogels can be chemically and biologically engineered by changing the amino acid sequence in order to enhance cell adhesion and cell proliferation [24,25]. Furthermore, by tailoring the peptide concentration, the hydrogels’ stiffness can be tuned allowing a precise control of the scaffold's final properties and the behaviour of cells cultured on them [26,27]. However, formulations leading to stiffer hydrogels may also limit the materials performance and/or the cell response. Hence, in the last decade, innovative approaches to reinforce hydrogels with different components used as “nanofillers” have been developed [12,[28], [29], [30]]. More recently, hydroxyapatite (Hap) nanoparticles have been used in tissue engineering studies as reinforcement to improve the scaffold's mechanical properties as well as cytocompatibility and bioactivity [31], [32], [33], [34]. Hap is the principal component of the mineral phase of bone, and it is also used as an extra scaffold component for bone regeneration [35]. Therefore, it is commonly used to mimic the natural bone due to its similarities in chemical composition, structure, and density [36]. Moreover, it has been shown that the nanotopography and chemical composition of the hydrogels are able to influence directly OC activity promoting OC differentiation, increasing cell number, and enhancing Tartrate-Resistant Acid Phosphatase (TRAP) activity [37,38].

For this reason, we have employed Hap nanopowder to create a bone-mimicking substrate to be used as a scaffold for successful culture and differentiation of OC precursors into OCs. We report here the incorporation of Hap within Fmoc-FF, Fmoc-serine (Fmoc-S) and Fmoc-arginyl-glycyl-aspartate (Fmoc-RGD) hydrogels to formulate Hap-containing, peptide-based hydrogel nanocomposites. We provide data on the characterization of these Hap-decorated hydrogels’ ultrastructure, mechanical properties under oscillatory rheometry, and cytocompatibility. Our results demonstrate that our formulated RGD-functionalised hydrogels can successfully incorporate Hap nanopowder to create a stiffer hydrogel microenvironment for OC cell culture. These hydrogel systems were shown to be biocompatible and are able to support cell adhesion and osteoclastogenesis through *in vitro* differentiation of Raw 264.7 pre-OC cells, as demonstrated by a typical OC morphology (presence of an actin ring, multinucleation, and ruffled plasma membrane) and increased TRAP expression. Thus, we envisage that this system could be used as a new biomaterial platform to culture and successfully differentiate OCs for tissue engineering applications, and ultimately provide a specialised scaffold to investigate the regulation of OC differentiation.

## 2 Materials and methods

### 2.1 Materials

Fmoc-FF/S (Fmoc-diphenylalanine/serine, 1:1 ratio) and Fmoc-FF/S/RGD (1:0.5:0.5 ratio) peptide powders were provided by Biogelx Ltd, UK. Peptide purity was assessed by Biogelx *via* High Performance Liquid Chromatography (HPLC). Fmoc-FF/S (commercial name Biogelx-S; Batch No. FFS052RM) and Fmoc-FF/S/RGD (commercial name Biogelx-RGD; Batch No. FFSRGD027RM) were 97% and 99% pure, respectively. Hydroxyapatite nanopowder (Ca_10_(PO_4_)_6_(OH)_2_, particle size < 200 nm), was obtained commercially (Sigma-Aldrich, 677418) and used as received.

### 2.2 Hydrogel and hap-decorated hydrogels preparation

Peptide hydrogels were prepared according to the manufacturer's protocol. In order to obtain a final hydrogel concentration of 15 mM, 0.0132 g of Fmoc-FF/S and 0.0148 g of Fmoc-FF/S/RGD peptide powder were dissolved in 1 mL of sterile deionized H_2_O to form a viscous peptide solution referred to as “pre-gel”. For the preparation of hydroxyapatite-containing hydrogels, 1 mg of Hap nanopowder was dispersed in 1 mL sterile deionized H_2_O using an ultrasonic water bath sonicator for 2 min at room temperature. This solution was then used to further dissolve the peptide powders using the above-mentioned ratios. When used for cell culture applications, “pre-gel” solutions were sterilized under UV light for 20 min prior hydrogel crosslinking induced by addition of high glucose Dulbecco's Modified Eagle's Medium (with 4.5 g/L glucose, L-Ala-L-Gln, sodium pyruvate and sodium bicarbonate) (DMEM, Sigma-Aldrich, FG0445, UK).

### 2.3 Atomic force microscopy (AFM)

Hydrogels with and without Hap were diluted from 15 mM to 5 mM using double deionised water (ddH_2_O). Samples for AFM analysis were prepared by depositing 100 µL aliquots of diluted hydrogel solutions onto freshly cleaved mica (Agar Scientific, UK) for 60 s at room temperature and allowing them to adhere. Excess liquid was then removed by capillary action using Whatman No. 1 filter paper. Hydrogel-coated mica samples were finally washed five times using 200 µL of ddH_2_O and left to air-dry overnight before imaging. Imaging was performed using a Bruker Multimode 8 AFM with a NanoScope V controller and a "J" scanner, operating under the NanoScope Controller software (v 8.15) (Bruker, USA). Scanning was performed in air at room temperature in ScanAsyst^TM^ (Peak Force Tapping) mode, using ScanAsyst-Air^TM^ probes (Bruker AXS S.A.S, France) with nominal resonant frequency (*f*_0_) and spring constant (*k*) of 70 kHz and 0.4 Nm^−1^ respectively. AFM images (2 µm^2^) were acquired with a 512 × 512 pixels resolution at a scan frequency of 1 Hz. Height data was first-order flattened, and average fibre/nanopowder widths were determined and analysed using the NanoScope Analysis software (v 1.40) (Bruker, USA). For this experiment, *N*=200 fibres were counted as a measure of homogeneity of the hydrogels structure.

### 2.4 Oscillatory rheometry

The viscoelasticity of the hydrogels, with and without Hap, was measured on a Malvern Kinexus Pro rheometer using a 20 mm/diameter parallel-plate geometry with a 0.5 mm gap size. Samples were prepared by pipetting 300 µL of pre-gel solution into a 24-well plate containing 1 mL/well of DMEM cell culture medium to form a spheroid-shaped hydrogel. After incubation for 2 h, fully formed hydrogels were transferred onto the rheometer plate. The elastic and viscous moduli of the hydrogels were recorded as a function of frequency between 0.1 and 100 Hz (shear strain of 0.1 %), at 25 °C.

### 2.5 Raw 264.7 hydrogel cell culture

Murine macrophage Raw 264.7 cells (TIB-71™) were purchased from ATCC and maintained in monolayer culture using DMEM containing 10% (v/v) foetal bovine serum (FBS) and 5% (v/v) Penicillin-Streptomycin-Amphotericin antibiotic mixture (PSA, 100 units/mL penicillin, 100 µg/mL streptomycin, 0.25 µg/mL amphotericin) (Sigma-Aldrich, UK). Upon reaching 70-80% confluency, cells were gently detached from the tissue culture flask by scraping and the cell suspension was pelleted by centrifugation (400 × *g* for 5 min). After counting cells with an automated cell counter (Countess™ 3, Invitrogen™), fresh culture medium was added to obtain the desired cell density. Hydrogels with and without Hap for cell culture were prepared 24 h in advance following the procedure described above for the pre-gel solution, followed by pipetting 200 µL of such pre-gel solution into the inner well of a 35 mm glass bottom dish for confocal microscopy (VWR, UK, 734-2905). 2 mL of DMEM were added to the pre-gel solution to induce gelation. The following day, DMEM used to crosslink the hydrogels was removed and 2 mL of cell suspension were added to the surface of the hydrogels to a final cell density of 4 × 10^5^ cell/mL. Medium was changed every 24 h for the first two days, and then every other day for up to 7 days.

### 2.6 F-actin staining: cell morphology

Morphology and cytoskeleton arrangement of Raw 264.7 cells cultured on Fmoc-FF/S and Fmoc-FF/S/RGD hydrogels, with and without Hap, were investigated using Alexa Fluor 488 Phalloidin (Invitrogen, UK, A12379). After 7 days in culture, medium was removed from the hydrogels and cells were fixed in 4% (w/v) paraformaldehyde (Sigma-Aldrich, UK) for 30 min and permeabilized in 0.5% (v/v) Triton X-100 solution (Sigma-Aldrich, UK) in phosphate buffered saline (PBS) (Gibco, UK) for 5 min. The samples were then incubated with 1:200 Alexa Fluor 488 Phalloidin for 30 min. After 3 × washes in PBS, nuclei were counterstained with 1:8000 Hoechst in PBS (Thermo Scientific, 33342, UK). Stained samples were imaged by using a Leica SP8 upright dipping lens confocal microscope with excitation filters of 495 nm (green, Alexa Flour) and 351 nm (blue, Hoechst). Cell analysis was performed by using ImageJ v. 1.51. In order to produce three 8-bit greyscale images, individual channels were obtained from the composite fluorescence images in red, green and blue (RGB). Greyscale images were thresholded using Huang's approach [39] and touching cells were separated into individual objects by applying a watershed algorithm [40]. Cell diameter and nuclei of at least 100 cells were measured by using the “analyse particle” plugin from ImageJ.

### 2.7 Scanning electron microscopy (SEM)

Hydrogel morphology and cell-hydrogel interaction were evaluated by Scanning Electron Microscopy (SEM). Briefly, 100 µL of the pre-gel solutions with and without Hap were pipetted into ThinCert well inserts (0.4 µm pore size Greiner Bio-One Ltd, UK). The inserts were then placed into 24-well plates and incubated at 37 °C with a total volume of 1.3 mL DMEM to fully crosslink the hydrogels. The following day Raw 264.7 cells were seeded onto the hydrogels as described above. After 7 days, cells were washed in PBS and fixed in 2.5 % (w/v) glutaraldehyde (Sigma-Aldrich, UK) and 4% (w/v) paraformaldehyde (Sigma-Aldrich, UK) in 0.1 M HEPES buffer (Sigma-Aldrich, UK). After rinsing the samples in PBS, for cell observation all samples were dehydrated in a graded ethanol (EtOH) series (25, 50, 75, 95, and 100 % v/v EtOH/water). Samples were maintained at 100 % EtOH and dried in a K850 Critical Point Drier (CPD, Quorum Technologies, UK). After the CPD step, samples were transferred into metallic pins and coated with gold palladium alloy using an SC7620 Mini Sputter Coater (Quorum). Samples were then imaged on a Quanta 250 FEG SEM (Thermo Fisher Scientific) at 20 kV.

### 2.8 Viability assessment

A Quant-iT™ PicoGreen™ dsDNA Assay Kit (Invitrogen P11496) was used to assess the viability of Raw 264.7 cells cultured on the Hap-decorated Fmoc-FF/S/RGD hydrogels. Cells were cultured into ThinCert well inserts as described above (Section 2.7). At each time point (24 h, 48 h, 5 days and 7 days), medium was removed from the inserts and the cell-cultured hydrogels were transferred into 1.5 mL Eppendorf tubes by gently peeling off the membrane of the ThinCert inserts. Then, 200 µL of 10 mg/mL Pronase in ddH_2_O (a commercial mixture of proteases from *Streptomyces griseus*, Roche, UK) were added to each Eppendorf tube and the mixtures vortexed for 20 s following an incubation of 5 min in a water bath at 37°C. An equal volume of 2 × TE buffer (20 µM Tris-HCl, 2 mM EDTA, 0.4% Triton X-100, pH 7.5; Sigma-Aldrich, UK) was added to each sample in order to extract the dsDNA. 100 µL of the dsDNA were then pipetted into a 96-well plate where an equal volume of PicoGreen Reagent (200-fold dilution in 1 × TE buffer) was added. After 5 min of incubation at room temperature the fluorescence intensity was measured by using a BioTek^TM^ FLx800^TM^ microplate fluorescence reader (excitation wavelengths: 480-512 nm; emission wavelength: 520 nm). The fluorescence intensity measured was normalised using day 1 of culture as a baseline control. All measurements were performed at least 3 times for each time point to ensure reproducibility.

### 2.9 Live/Dead staining

A Live/Dead assay kit (Invitrogen L3224) was used to assess the viability of Raw 264.7 cells cultured on the Hap-decorated Fmoc-FF/S/RGD hydrogels. Following the manufacturer's protocol, 600 µL of the assay solution containing 4 µM ethidium homodimer-1 (EthD-1) and 2 µM calcein AM were pipetted onto the cell-hydrogel constructs. After 30 min of incubation cells were washed 3 times in PBS and imaged using a Nikon Eclipse 50i fluorescence microscope (emission wavelengths: green channel for live cells 515 nm; red channel for dead cells 635 nm; excitation wavelength: 495 nm). Cell images were collected at 24 h, 48 h, 5 days and 7 days post culture. Three images per time point were acquired.

### 2.10 TRAP immunofluorescence staining

Differentiation of Raw 264.7 cells into mature osteoclasts was determined by using a TRAP monoclonal antibody. After 7 days in culture on hydrogels, cells were stained following the procedure described in section 2.6 using 1:200 anti-TRAP mAb conjugated Alexa Fluor 594 (Santa Cruz Biotechnology, UK, sc-376875) in PBS for 90 min. After the incubation time, cells were washed three times for 5 min with PBS and imaged immediately using a Nikon Eclipse 50i fluorescence microscope (excitation wavelength 495 nm). Fluorescence intensity ratio was measured from 100 cells and normalised to the background by using ImageJ v. 1.51.

### 2.11 TRAP gene expression

Raw 264.7 cells were cultured in Hap-decorated Fmoc-FF/S/RGD hydrogels using ThinCert well inserts, as described above (Section 2.7). After 3 and 7 days post culture the hydrogels were removed from the inserts and transferred to 1.5 mL Eppendorf tubes. Then, 1 mL of TRIzol™ Reagent (Invitrogen, 15596026) was added to each hydrogel and RNA was extracted following the manufacturer's protocol. Quality and quantity were determined using a Nanodrop ND-1000 Spectrophotometer (Nanodrop Technologies). Following the RNA extraction, RNA samples were reverse-transcribed into cDNA using a High Capacity Reverse Transcription Kit (Applied Biosystems, UK). Obtained cDNA was further diluted to 5 ng/µL according to the Nanodrop reading and qPCR was performed in triplicate using a StepOne™ Real-Time PCR System (Applied Biosystems, UK) using TaqMan probes with the universal PCR Master Mix (Life Technologies, 4304437) in a total volume of 10 µL. The TaqMan probe for Trap was used (Mm00475698_m1) and data was analysed using the 2^−∆Ct^ method and normalised to the endogenous house-keeping gene GAPDH (Mm99999915_g1).

### 2.12 Statistical analysis

All quantitative values are presented as mean ± standard deviation. All experiments were performed using at least three replicates. Data were plotted using Origin 2019b and compared using an unpaired *t* test, unless stated otherwise. Two levels of significance were used: 0.005 (**) and 0.001 (***).

## 3 Results and discussion

### 3.1 Formulation and characterization of the Hydroxyapatite-decorated self-assembled peptide hydrogels

**Fig. 1:**
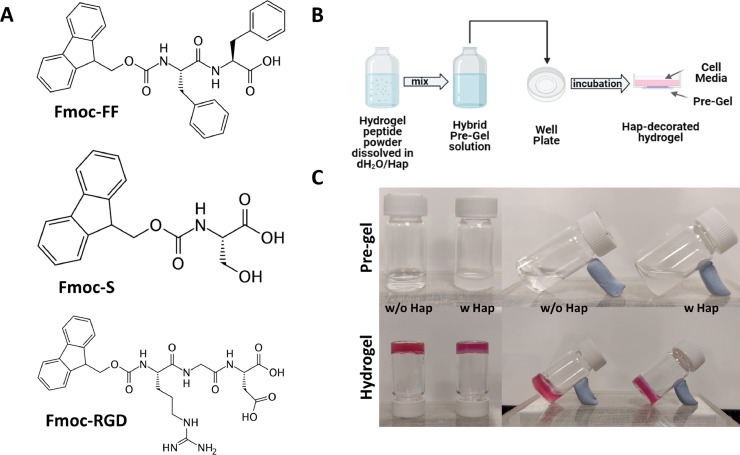
Hap-decorated hydrogels protocol development. (A) Molecular structure of the three building blocks Fmoc-FF, Fmoc-S, Fmoc-RGD. The Fmoc-FF/S is made from Fmoc-FF and Fmoc-S. The Fmoc-FF/S/RGD hydrogel is made from these two peptides plus Fmoc-RGD. (B) Schematic representation of the optimised protocol used to incorporate the Hap nanonpowder within hydrogels. (C) Pre-gel and hydrogel vials photograph w/wo the addition of Hap.

In order to develop multicomponent hydrogels for use as scaffolds for OC culture and differentiation, Fmoc-FF/S and Fmoc-FF/S/RGD were used. These peptide-based formulations form hydrogels through cooperative assembly of the short peptides Fmoc-FF, Fmoc-S and Fmoc-RGD (Fig. 1**A**), and have been modified here by the incorporation of Hap nanopowder as “nanofiller” in the hydrogel network. It has been demonstrated that modifying scaffolds with calcium phosphate minerals makes them a suitable option for use in bone regeneration studies by increasing their mechanical properties, cytocompatibility and bioactivity [30]. Here, we developed a protocol through which Hap nanoparticles were successfully incorporated within hydrogel networks, without impairing the gelator assembling mechanism (Fig. 1**B**). Hap nanoparticles were successfully incorporated in both hydrogel formulations by using a viscous liquid dispersion that formed a hybrid gel-like solution when added to the peptide hydrogels (Section 2.2). Different concentrations of Hap nanoparticles (up to 3 mg/ml) were tested. However, Hap, at a concentration of 1 mg/mL, was chosen for this study as it provided the best mechanical properties among all the formulated nanocomposite (**Fig. ESI 1**). Undecorated pre-gels were clear and transparent, while hybrid pre-gel solutions appeared cloudy, due to the presence of the Hap dispersion (Fig. 1**C**). All the hydrogels were formed by addition of DMEM, which acts as a source of Ca^2+^ ions, crosslinking the carboxylate groups of the peptide's C-termini [26]. Resulting hydrogels were stable with and without (w/wo) the presence of Hap, as shown by the glass vial flip test (Fig. 1**C**). In particular, Fmoc-FF/S/RGD showed a homogeneous distribution of Hap throughout the hydrogels with minimal leaching over time (**Fig. ESI 2**).

**Fig. 2:**
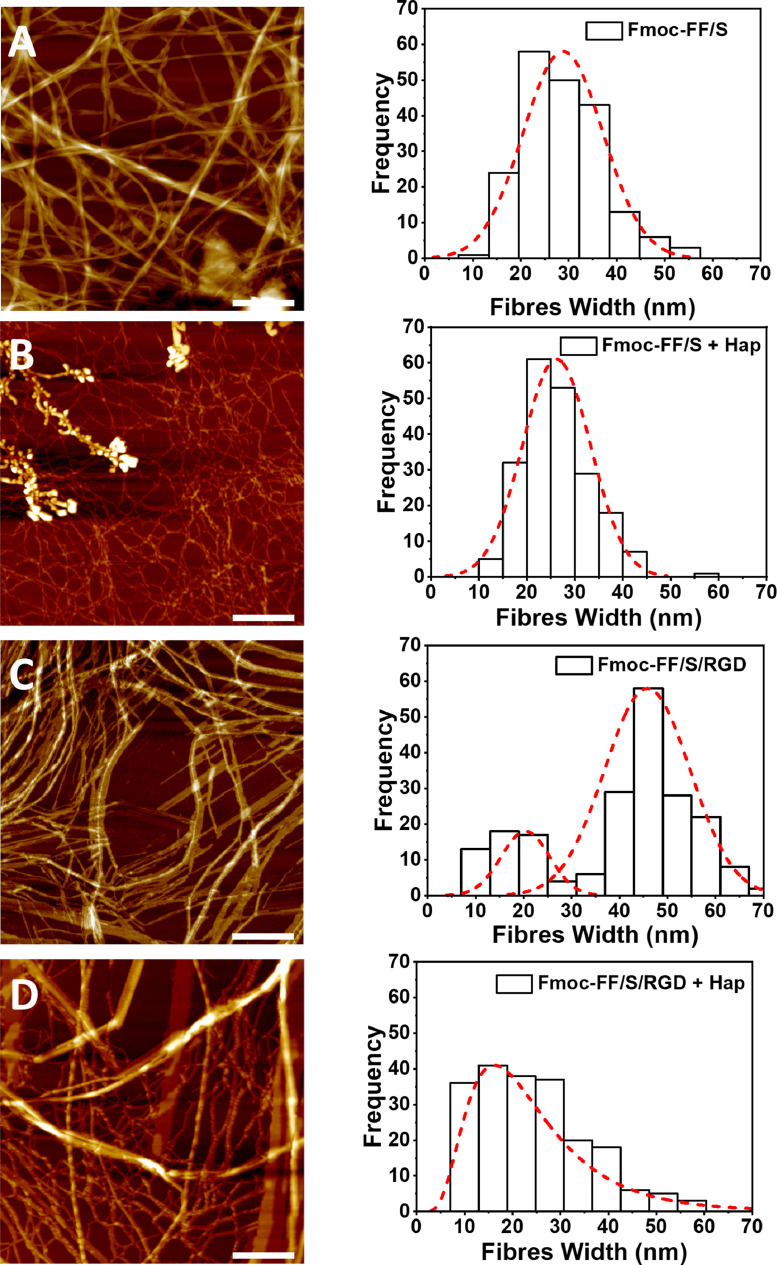
AFM images of the hydrogels studied here, and distributions of fibre widths as measured on the AFM images (A-D). Hydrogel nanotopography of Fmoc-FF/S (A) and Fmoc-FF/S/RGD (C) without added Hap, and Fmoc-FF/S (B) and Fmoc-FF/S/RGD (D) after incorporation of Hap (Scan size 2 µm^2^, Scale bar 400 nm).

Once formulated, the microstructure of each hydrogel was assessed using AFM (Fig. 2**)**. To investigate the effect of Hap addition, two different samples of each hydrogel formulations were prepared w/wo Hap. All four samples showed the typical hydrogel nanofibre morphology with long bundles and extensive fibre entanglement, suggesting that the addition of Hap did not cause impairment of the peptide self-assembling mechanism nor any fibre precipitation (Fig. 2**A–D**). Fibre width analysis of the Fmoc-FF/S hydrogels w/wo Hap (Fig. 2**A,B**) shows unimodal distributions in both cases, with average fibre widths of 26 (±7) nm (with Hap) and 29 (±9) nm (without Hap). In contrast, the Fmoc-FF/S/RGD hydrogel without Hap shows a bimodal distribution, with a first peak centred at 19 nm and a second, more populated distribution with peak centred at 44 nm (Fig. 2**C**). However, after incorporation of Hap the Fmoc-FF/S/RGD hydrogel shows a higher number of thinner fibres (diameter ∼10 nm) and a broad unimodal distribution with marked positive skewness and a peak at 12-15 nm (Fig. 2**D**).

**Fig. 3:**
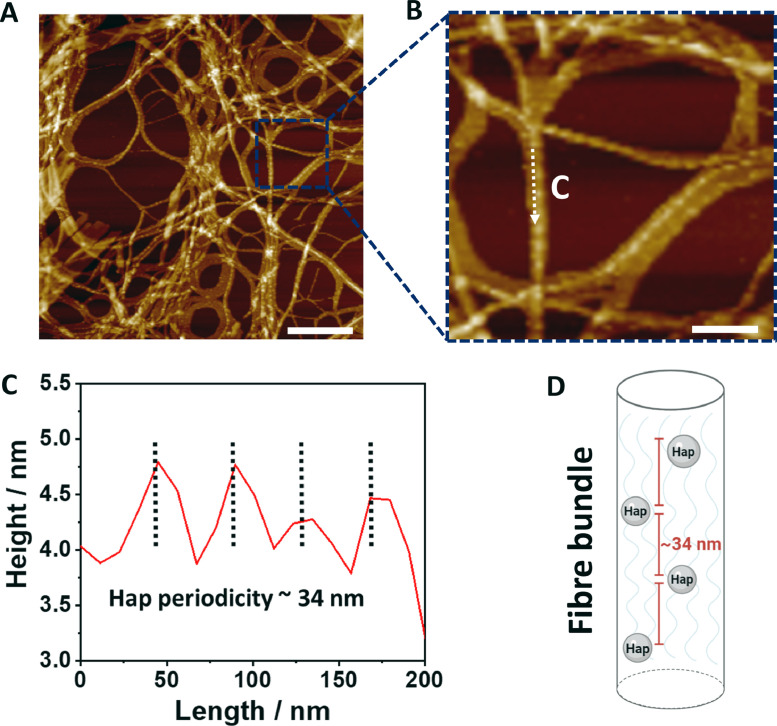
Topography of Hap-decorated Fmoc-FF/S/RGD hydrogels. (A) 2 µm^2^ AFM images of Fmoc-FF/S/RGD incorporating Hap in a repeating pattern. (B) Enlarged AFM image of the area outlined in A showing Hap nanoparticles decorating the Fmoc-FF/S/RGD fibres. (C) Corresponding height profile of the linear scan indicated by the white arrow in B. (D) Schematic model of a hydrogel fibre incorporating Hap. Scale bar for A is 400 nm. Scale Bar for B is 200 nm.

Based on these results, there is little or no difference in fibre width distribution between the Fmoc-FF/S hydrogels w/wo Hap (Fig. 2**A-B**). We believe that this is due to the lack of interaction between Hap and the two monomers present in the hydrogel, Fmoc-FF and Fmoc-S (Fig. 1**A**) [41,42]. For similar reasons it is plausible to postulate that the different width distribution observed in Fmoc-FF/S/RGD AFM images (Fig. 2**C**) is due to the presence of the Fmoc-RGD peptide within the hydrogel structure. Although it does not impair the self-assembly mechanism of the hydrogels, introduction of a third monomer (Fmoc-RGD) causes a bimodal distribution with two populations of thinner and thicker fibres, probably due to steric interference from the RGD groups that may impede fibre-fibre lateral associations, as shown in a similar peptide-based hydrogel system by Green and co-workers [43]. However, based on the width distribution shown in Fig. 2**D** we hypothesise that the incorporation of Hap nanoparticles to Fmoc-FF/S/RGD hydrogels may induce the peptide nanofibres to form thinner structures, in order to “accommodate” such Hap nanoparticles within the fibrillar structure. As demonstrated by Zhou *et al.*, Fmoc-FF and Fmoc-RGD peptide sequences are able to self-assemble into cylinder-like structures with the RGD motifs present on the fibre surfaces [25]. For this reason, we believe that, during the hydrogel self-assembling mechanism, the presence of Hap in the system “forces” the formation of thinner fibres, due to steric interaction. In fact, it has been shown that the presence of nanoparticles on fibre surfaces decreases the mobility of monomers causing a slow conformational relaxation of the polymers in fibres [44]. Furthermore, we found that, when Hap was incorporated into the Fmoc-FF/S/RGD hydrogels, the Hap nanoparticles appeared to be arranged on the peptide nanofibres in a regular pattern (Fig. 3**A**). Indeed, the AFM height measurements showed a periodicity of ∼34 nm between the height peaks of the Hap nanoparticles along the fibre axis (Fig. 3**B-C**). Hap nanoparticle size distribution ranges between ∼ 5 and 25 nm and a tighter distribution of nanoparticles on the fibres can be observed (**Fig. ESI 3A,B**). It can therefore be proposed that the repeated pattern shown in Fig. 3**A-C** is due to the Hap nanoparticles decorating the nanofibres at each point of periodicity. It has been demonstrated that polar and positively charged amino acids (such as Arginine, Arg) can strongly interact with and adsorb Hap nanoparticles [42,45]. Due to the presence of positively charged Arg side chains in the exposed RGD motifs on the peptide nanofibre surface, we hypothesise that Hap nanoparticles are interacting with the peptide nanofibres, resulting in a more homogenous incorporation of Hap nanoparticles in this hydrogel compared to Fmoc-FF/S, which lacks the RGD motif (Fig. 2**A-B**). In particular, due to the electrostatic attraction between Arg and Hap (positively and negatively charged at formulation), it is likely that the Hap nanoparticles are able to “coat” the RGD-containing nanofibres to successfully form hybrid hydrogels, as schematically proposed in Fig. 3**D**. In fact, we believe that, similar to what shown by Cerruti and colleagues [46], the RGD motif present here exposes both positively (Arg) and negatively (Asp) amino acid side chains and these may be involved in large-range electrostatic interactions (Fig. 3**A**) [42,45,47].

#### 3.1.1 Mechanical characterization of the hydrogels

**Fig. 4:**
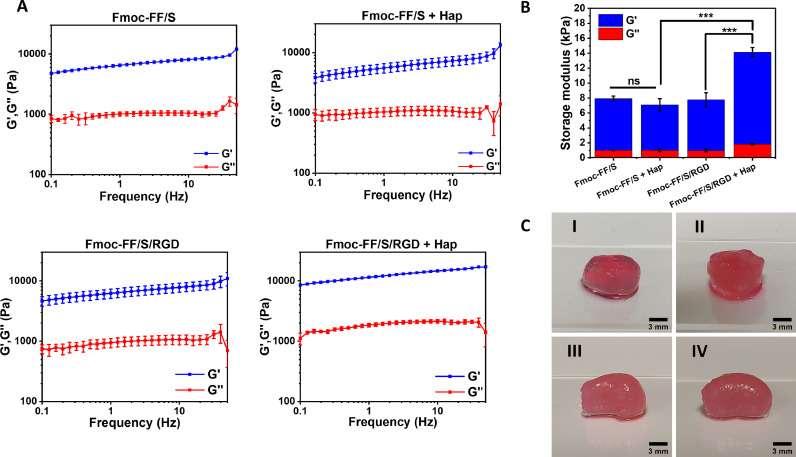
Analysis of the mechanical properties of the hydrogels studied here. (A) Frequency sweep rheology measurments of Fmoc-FF/S and Fmoc-FF/S/RGD with and without Hap (0.1-100 Hz 0.1 % strain). (B) Comparison of the storage moduli at 1 Hz, 0,1% strain of the Fmoc-FF/S and Fmoc-FF/S/RGD hydrogels with and without Hap. (C) Photographs of the spheroids of Fmoc-FF/S (I), Fmoc-FF/S+Hap (II), Fmoc-FF/S/RGD (III) and Fmoc-FF/S/RGD+Hap (IV) used for the rheology experiments. All measurements were performed at least 3 times at 25 °C. (Data are shown as mean ± SD; ***p-value <0.001; **p-value <0.05).

In order to assess the effect of Hap incorporation on the hydrogels’ mechanical properties, we analysed by rheology the viscoelastic properties of the Hap-decorated versus undecorated hydrogels. Both hydrogel formulations Fmoc-FF/S and Fmoc-FF/S/RGD, w/wo Hap, were subjected to a frequency sweep experiment (frequency range 0.1 to 100 Hz, strain 0.1 %). As shown in Fig. 4**A**, all hydrogels showed a wide linear viscoelastic region (LVR) with the storage modulus (*G’*) consistently higher than the loss modulus (*G’’*) throughout the entire frequency range. This result indicates that both Fmoc-FF/S and Fmoc-FF/S/RGD hydrogels were able to incorporate Hap while still forming a solid-like, hydrogel structure (Fig. 4**C**). The value of *G´* (used as an indicator of hydrogel stiffness) could be modulated according to the formulation used. The two undecorated hydrogels Fmoc-FF/S and Fmoc-FF/S/RGD show similar values in storage modules (6.92 ± 0.33 vs 6.79 ± 0.95 kPa, respectively), indicating that the addition of Fmoc-RGD does not change significantly the mechanical properties between these two hydrogels. Incorporation of Hap to Fmoc-FF/S resulted in apparent 1.2-fold decrease in storage modulus (6.92 ± 0.33 vs 6.07 ± 0.82 kPa, respectively) but the differences were not significant (*p*-value = 0.8958) (Fig. 4**B**). On the other hand, addition of Hap to Fmoc-FF/S/RGD resulted in hydrogels with a significant increase in storage modulus compared to both the undecorated Fmoc-FF/S/RGD hydrogels (12.28 ± 0.61 vs 6.79 ± 0.33 kPa, respectively *p* < 0.005) and the Hap-decorated Fmoc-FF/S hydrogels (12.28 ± 0.61 vs 6.07 ± 0.82 kPa, respectively *p* < 0.001, Fig. 4**B**).

Our findings also support those of Ghosh and colleagues [42], who demonstrated that Arg-presenting Fmoc-based hydrogels can incorporate Hap nanoparticles to make stiffer scaffolds. In particular, the increase in stiffness occurring in the Fmoc-FF/S/RGD hydrogel when incorporating Hap is greater than for the Fmoc-FF/S hydrogel due to the presence of exposed Arg side chains in the peptide-hydrogel structure. Moreover, in line with our AFM results, it is reasonable to postulate that electrostatic interactions between Arg and Asp side chains from Fmoc-RGD and Ca^2+^ and PO_4_^3−^ ions from Hap will increase the local supersaturation of Hap nanoparticles, induce a higher number of nanofibre entanglements and fibre-fibre interactions, and will result in hydrogels with superior mechanical properties compared to the hydrogels without either RGD or Hap [46].

### 3.2 Cytocompatibility and cell morphology analysis of the Hap-decorated hydrogels

**Fig. 5:**
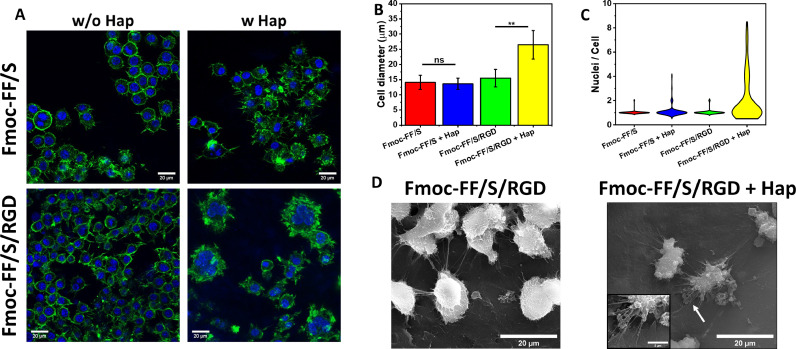
Analysis of Raw 264.7 cell culture and differentiation on 15 mM Fmoc-FF/S and Fmoc-FF/S/RGD hydrogels with and without incorporated Hap. (A) F-actin staining of Raw 264.7 cells after seven days culture on the different hydrogels (green: F-actin, Alexa Fluor 488 phalloidin; blue: Nuclei, Hoechst 33342; scale bars 20 µm). (B) Measurements of cell diameter (data shown as mean ± SD, N=43, **p <0.05). (C) Analysis of multinucleation by a violin distribution plot (data shown as number of nuclei per cell, N=100). (D) SEM images of Raw 264.7 cells cultured on Fmoc-FF/S/RGD with or without incorporated Hap. White arrow shows elongated pseudopodia of Raw 264.7-derived OC. (Data are shown as mean ± SD; ***p-value <0.001; **p-value <0.05).

The cytocompatibility of the newly developed Hap-decorated scaffolds was examined using Raw 264.7 cells, a murine pre-osteoclast (pre-OC) cell line that is a well-established model to assess OC differentiation and bone-resorbing capability [48]. Three main OC features were used to assess their differentiation: presence/absence of an actin ring, multiple invagination of the cell membrane (hereafter referred to as a ruffled border) and more than three nuclei per cell. As shown in Fig. 5**A**, an actin ring surrounded all cells in every hydrogel formulation tested, regardless of the presence of Hap. However, when cells were cultured on Fmoc-FF/S/RGD with Hap, they showed a clear difference in morphology: cells appeared well spread with a prominent ruffled border. Moreover, Raw 264.7 cell size significantly increased on the Fmoc-FF/S/RGD hydrogels in the presence of Hap (Fig. 5**B**); the mean diameter showed that the cells were larger (26.5 ± 4.7 µm) than their counterparts cultured on either the undecorated hydrogels (15.5 ± 2.9 µm) or Fmoc-FF/S with (13.6 ± 1.9 µm) and without Hap (14.1 ± 2.3 µm) (Fig. 5**B**).

Furthermore, there was evidence of multinucleation on Raw 264/7 cells cultured on Fmoc-FF/S/RGD with incorporated Hap, suggesting OC differentiation (Fig. 5**A**). A violin plot of the number of nuclei per cell (Fig. 5**C**) shows that the majority of cells had a single nuclei irrespective of the hydrogel substrate. However, when cultured on Fmoc-FF/S/RGD with incorporated Hap, Raw 264.7 cells showed up to 9 nuclei/cell with a higher number of cells with 4-8 nuclei. This distribution was significantly different from that seen for cells cultured on the other hydrogels (*p* < 0.0001, non-parametric ANOVA). Cell morphology was also analysed by SEM. Fig. 5**D** shows differences in morphology of Raw 264.7 cells cultured on Fmoc-FF/S/RGD w/wo Hap. Cells appear rounded and smaller in diameter in the absence of Hap. On the other hand, when cultured in the presence of Hap, cells show a well spread morphology and are larger in diameter, with orientated pseudopodia (Fig. 5**D**). Interestingly, these pseudopodia spread all around the cell body and are used to create a solid focal adhesion, tightly connected with the hydrogel substrate (Fig. 5**D**). We believe that two major factors are involved in the observed differentiation of Raw 264.7 cells into OCs when cultured on Fmoc-FF/S/RGD hydrogels with incorporated Hap. The first factor is the presence of the RGD motif in the hydrogel's peptide composition. When binding to bone, OCs form an actin ring-mediated sealing zone that is mediated in part by integrins which are located on the cell membrane and interact with the surrounding ECM. The most important integrin in OC is αvβ3, which strongly interacts with the RGD motif [6,49]. It is known that depletion αvβ3 can cause an impairment in OC differentiation and function [50,51]. Hence, the presence of RGD motifs allows Raw 264.7 cells to adhere better to the Fmoc-FF/S/RGD hydrogels. However, differentiation toward mature OCs only occurred when the Hap was incorporated within the hydrogels. Therefore, our data suggests that the presence of Hap is the second crucial factor for OC-differentiation. Indeed, it has been previously demonstrated that Hap has an effect on pre-OC cells, enhancing the formation of OCs with a pronounced actin ring, protruding pseudopodia and prominent ruffled border [52], [53], [54], similar to the features observed here.

### 3.3 Hydroxyapatite-decorated hydrogels as substrate for osteoclast culture and differentiation

**Fig. 6:**
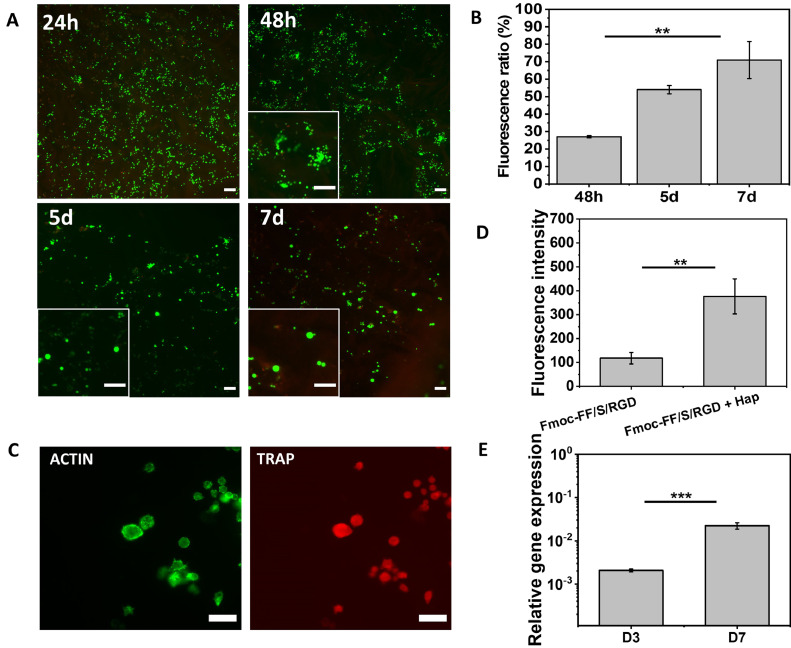
Hap-decorated Fmoc-FF/S/RGD hydrogel functionality. (A) Analyis of viability of Raw 264.7 cells cultured on Fmoc-FF/S/RGD+Hap at different time points using a Live/Dead assay (green: viable cells, calcein AM; red: dead cells, ethidium homodimer-1; scale bar 100 µm). (B) Quantification of dsDNA of Raw 264.7 cells cultured on Fmoc-FF/S/RGD+Hap at different time points, using a PicoGreen fluorescence assay (values normalised to 24 h as baseline control). (C) Visualisation of TRAP expression by immunofluorecence staining (green: F-actin, Alexa Fluor 488 phalloidin; red: TRAP, anti-TRAP mAb conjugated Alexa Fluor 594). (D) Relative TRAP immunofluorescence intensity of cells cultured on Fmoc-FF/S/RGD hydrogels with and without incorporated Hap (fluorescence intensity was measured from at least N=100 cells and corrected against the background). (E) Gene expression of TRAP relative to GAPDH by Raw 264.7 cells (n = 3) after three (D3) and seven (D7) days culture on Hap-decorated Fmoc-FF/S/RGD hydrogel. (Data shown as mean ± SD; ***p <0.001; **p <0.05).

Having demonstrated the potential of the Fmoc-FF/S/RGD with Hap incorporated system for the culture of pre-OC cells, we conducted additional functional assays to assess cell viability and OC-differentiation. By analysing cell viability we investigated whether Raw 264.7 cells proliferated on our system before differentiating into OCs. LIVE/DEAD staining showed mainly viable cells at 24 h and 48 h (Fig. 6**A**). Interestingly, from 48 h onward, cells start to form clusters (as shown in the higher magnification inset) and, after 5 days (5d), live cells appear to be larger in size (Fig. 6**A**, 5d–7d, higher magnification) with little or no clusters present. Furthermore, PicoGreen assay data showed a significant increase of dsDNA over time (*p* < 0.005), indicating a ∼70% cell proliferation with respect to day 0 baseline control (Fig. 6**B**).

According to these results, we believe that Raw 264.7 cells start their commitment to OC differentiation within 3 days post seeding onto the Fmoc-FF/S/RGD with incorporated Hap hydrogels, and at day 7 they eventually form OCs. For this reason, the increase of dsDNA seen at day 7 is likely to reflect a higher quantity of DNA expressed per cell due to the presence of multinucleated cells (as also shown in Fig. 5**A** and **C**), rather than an increase in cell number.

Finally, to confirm the differentiation of Raw 264.7 cells into OCs we assessed the expression of Tartrate-Resistant Acid Phosphatase (TRAP), the most common OC marker [55,56]. TRAP is a phosphatase used by mature OC to acidify the bone microenvironment during their resorption activity. Its expression is localised within the ruffled border area, the lysosomes and the Golgi cisternae [57]. Thus, firstly, we confirmed the expression of this OC marker by using an anti-TRAP monoclonal antibody staining. Raw 264.7 cells cultured on Hap-decorated Fmoc-FF/S/RGD gels showed a positive, bright expression of TRAP localised within the cell membrane (Fig. 6**C**). Its expression was significantly higher (*p*<0.005) when compared to Fmoc-FF/S/RGD without Hap (Fig. 6**D**). Moreover, qRT-PCR was also used to confirm the expression of TRAP when cells were cultured on Fmoc-FF/S/RGD with incorporated Hap. Indeed, since the OC-commitment it is known to begin around days 2–3 [5], we compared the expression of TRAP at day 7 with that at day 3 and we successfully demonstrated that there was a significant increased expression of TRAP at day 7 (Fig. 6**E**), confirming the OC-differentiation of Raw 264.7 cells cultured on Fmoc-FF/S/RGD with incorporated Hap. Taking into consideration the changes in Raw 264.7 morphology, the increase of multinucleation, and the increase in expression of the OC-typical marker TRAP, we can conclude that our newly developed Hap-decorated hydrogel system is able to support osteoclastogenesis *in vitro*.

## 4 Conclusions

In this work, we successfully formulated a Hap-decorated Fmoc-based peptide hydrogel that could be used as a bone-mimicking scaffold for 2D culture and differentiation of OC cells. Our material characterization indicates that the Hap decorates the peptide hydrogels, possibly through a physiochemical interaction between the Fmoc-RGD-functionalised peptide nanofibres and the Hap surface. In particular, it is likely that an attractive electrostatic interaction occurs between the exposed side chains from the RGD motif present on the peptide nanofibres and the Hap nanopowder surface. In fact, both the Arg and Asp side chains of the RGD peptide are likely to participate in the binding of the Hap surface to the Fmoc-FF/S/RGD hydrogel. Molecular dynamics simulations suggest that electrostatic interactions play a dominant role in the binding of peptides and proteins to Hap surfaces and that charged groups such as carboxylate, amine or guanidine may interact with the calcium, phosphate and hydroxide ions on the Hap crystal lattice [58], [59], [60]. This could explain the increased mechanical properties (raise of G') observed for Fmoc-FF/S/RGD incorporating Hap compared to the other hydrogels, as shown in Fig. 4**A**. Our findings are consistent with published data showing that hydrogels based on amino acids with ionic side chains can incorporate Hap nanoparticles to make stiffer scaffolds, probably by forming strong interactions with Ca^2+^ and PO_4_^3−^ ions coming from dissolved Hap [41,61,62,42]. The Hap-decorated Fmoc-FF/S/RGD hydrogels show potential as platforms to culture and differentiate Raw 264.7 cells toward mature OCs. In particular, the presence of both RGD motifs and Hap within these hydrogels supported the viability of cultured Raw 264.7 cells over time and triggered their differentiation into mature OCs. This differentiation was confirmed by changes in cellular morphology (significant increase in multinucleation, ruffled border, and cell diameter) and by increased expression of TRAP, a typical OC marker [55]. Unlike other different hydrogel systems for culturing OC (such as gellan gum, alginate or hyaluronic acid hydrogels [63], [64], [65]), our system resembles the typical OC-bone interface. In fact, despite its limitations (e.g. 2D platform, lack of collagen component, mechanical properties not as stiff as native tissue), due to the presence of the RGD motif, OCs interact with a biomineralised peptide surface in an active way through typical RGD-integrin binding (integrin α_v_β_3_) [51]. Importantly, though, our functionalised hydrogel supports osteoclastogenesis *in vitro* and does not require the presence of inducing factors such as RANKL [66] to generate mature OCs. All these results suggest that the Hap-decorated hydrogels developed here could provide a material platform to design more complex and translatable biomaterials for OC culture and differentiation. Moreover, such hydrogels may help to investigate and develop novel *in vitro* models for bone regeneration/resorption studies. For example, this system could be loaded with drugs that inhibit OC differentiation [67] to develop more efficient pharmacological treatments to tackle excessive bone degradation, as it occurs in osteoporosis, osteoarthritis, and cancers.

## Declaration of Competing Interest

The authors declare the following personal relationships which may be considered as potential competing interests: At the time of submission, CA was employed by Biogelx Ltd.

## Appendix Supplementary materials

Image, application 1
