# Supplementary material for: Hydroxyapatite-decorated Fmoc-hydrogel as a bone-mimicking substrate for osteoclast differentiation and culture
Source: Acta Biomater. 2022 Jan 15;138:144–54. doi: 10.1016/j.actbio.2021.11.011 (PMC8756142; doi:10.1016/j.actbio.2021.11.011)
Supplement: Supplementary file 1 [file mmc1.docx]

**Hydroxyapatite-decorated Fmoc-hydrogel as a bone-mimicking substrate for osteoclast differentiation and culture**

Mattia Vitale ^a^, Cosimo Ligorio ^a^, Bethan McAvan ^a^, Nigel W. Hodson ^b^, Chris Allan ^c^, Stephen M. Richardson ^a*^, Judith A. Hoyland ^a*^_,_ Jordi Bella ^a*^

**Electronic Supplementary Information**

**
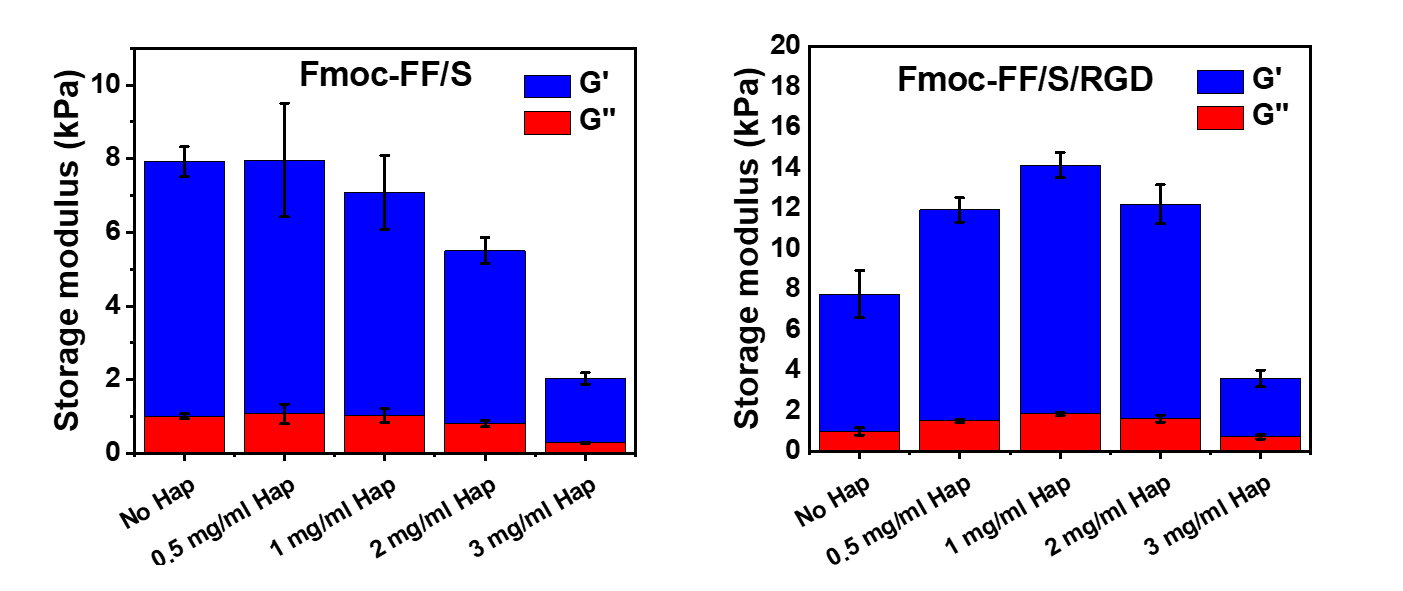
**

**Figure ESI 1:** Storage moduli at 1 Hz, 0,1% strain of Fmoc-FF/S and Fmoc-FF/S/RGD with different concentration of Hap nanoparticles. Addition of Hap up to 3 mg/ml to Fmoc-FF/S showed a detrimental effect on G’ (~3.94 fold decrease compared to the undecorated hydrogel). Similar behaviour was observed for Fmoc-FF/S/RGD when up to 3 mg/ml of Hap were added (~ 2.34 fold decrease compared to the naked hydrogel). Only Fmoc-FF/S/RGD decorated with 1 mg/ml Hap showed a significantly increased storage modulus among the Hap concentration tested. For this reason, 1mg/ml Hap was used for both hydrogels formulation (Fmoc-FF/S and Fmoc-FF/S/RGD) as final concentration for this study.

**
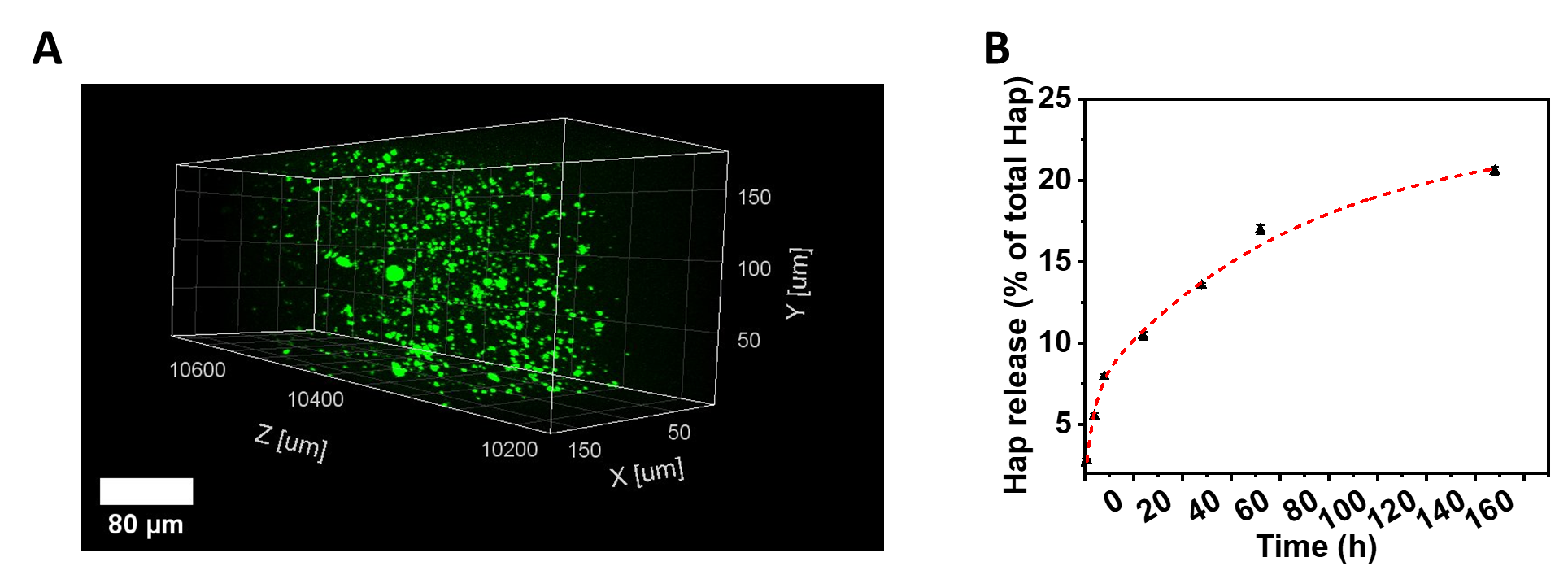
**

**Figure ESI 2: A)** Image stack of Fmoc-FF/S/RGD hydrogel showing calcein-stained Hap (conc. 1mg/ml) homogenously distributed within the gel network. Fluorescent calcein-stained Hap has been obtained following the method described by Hale *et al.* [1]. Briefly, 1mL aliquot of 1 mg/ml Hap (stock solution 5 mg/ml) was incubated with 1 µL of Calcein AM (4µM, Invitrogen L3224) for 2 h at 4°C, under constant agitation. After incubation, the Hap/calcein was washed 3x with 1mL dH_2_O and incorporated within the hydrogel following the procedure described in section *2.2 Hydrogel and Hap-decorated hydrogels preparation*. Stained samples were imaged by using a Leica SP8 upright dipping lens confocal microscope with excitation filters of 495 nm (green, Alexa Flour) and 351 nm (blue, Hoechst). Image analysis was performed by using Imaris cell analysis software (v.9.8). **B)** Cumulative release fit of Hap from Fmoc-FF/S/RGD hydrogel over time. Data were obtained using the same method described above to label the Hap nanoparticles.


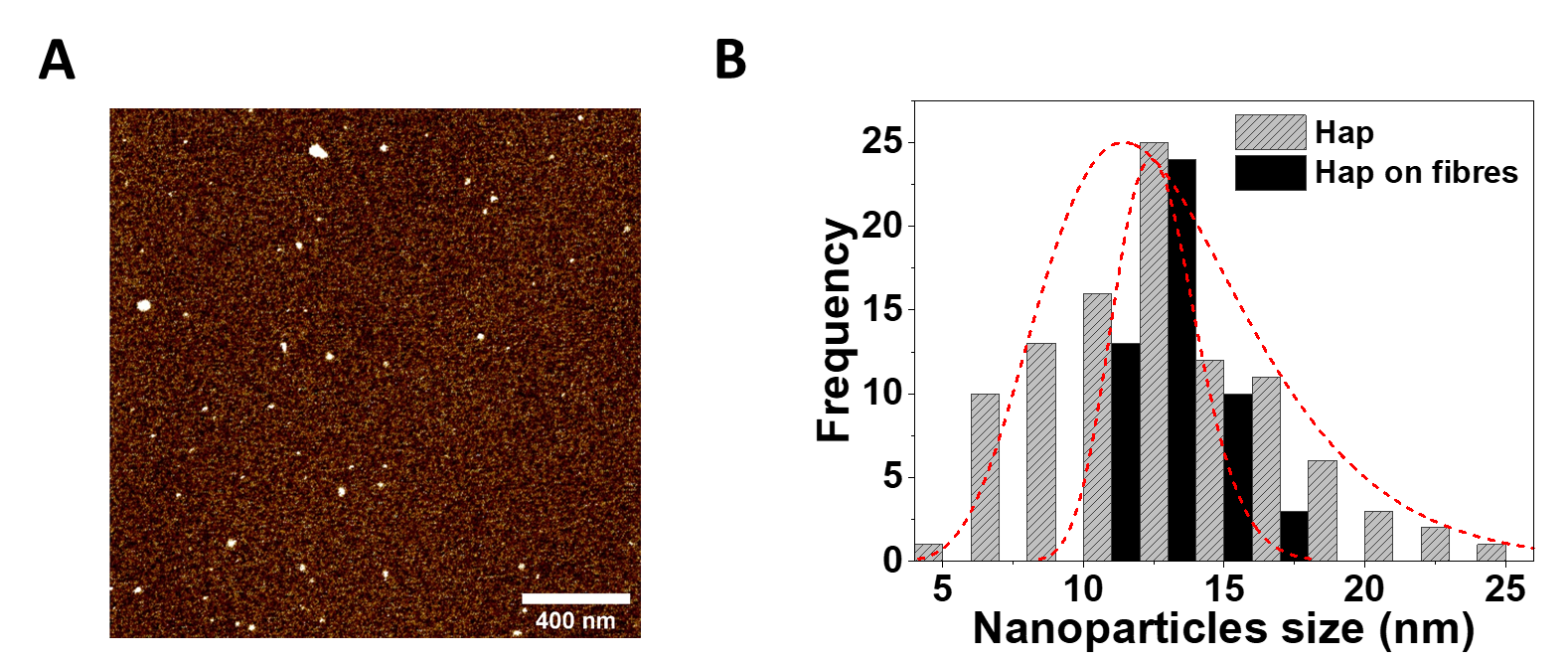


**Figure ESI 3: A)** AFM image of Hap nanoparticles (*conc. 1 µg/ml*) in a 2 µm^2^ scan size region on polylysine–coated mica. **B)** Hap nanoparticles size distribution alone (grey bars) and on the Fmoc-FF/S/RGD hydrogel fibres (black bars)**.** White arrows point to Hap aggregates.

**References**

[1] L. V Hale, Y.F. Ma, R.F. Santerre, Semi-quantitative fluorescence analysis of calcein binding as a measurement of in vitro mineralization, Calcif. Tissue Int. 67 (2000) 80–84. https://doi.org/10.1007/s00223001101.
